# Combined HRAS and NRAS ablation induces a RASopathy phenotype in mice

**DOI:** 10.1186/s12964-024-01717-4

**Published:** 2024-06-17

**Authors:** Rocío Fuentes-Mateos, Rósula García-Navas, Cristina Fernández-Infante, Luis Hernández-Cano, Nuria Calzada-Nieto, Andrea Olarte-San Juan, Carmen Guerrero, Eugenio Santos, Alberto Fernández-Medarde

**Affiliations:** 1 Centro de Investigación del Cáncer-Instituto de Biología Molecular y Celular del Cáncer (CSIC-Universidad de Salamanca) and CIBERONC, Campus Unamuno, University of Salamanca, 37007 Salamanca, Spain; 2 Instituto de Biología Molecular y Celular del Cáncer (IMBCC), USAL-CSIC. Instituto de Investigación Biomédica de Salamanca (IBSAL), Salamanca, Spain; 3 Departamento de Medicina, Universidad de Salamanca, Salamanca, Spain; 4 Present address: Department of Molecular Pharmacology, Groningen Research Institute for Asthma and COPD, University of Groningen, Groningen, Netherlands; 5 Present address: Department of Nuclear Medicine and Molecular Imaging, University Medical Center Groningen, Groningen, Netherlands

**Keywords:** RASopathy, RAS isoforms, HRAS;NRAS ablation, Developmental disorders, Thrombocytopenia

## Abstract

**Background:**

HRAS^KO^/NRAS^KO^ double knockout mice exhibit exceedingly high rates of perinatal lethality due to respiratory failure caused by a significant lung maturation delay. The few animals that reach adulthood have a normal lifespan, but present areas of atelectasis mixed with patches of emphysema and normal tissue in the lung.

**Methods:**

Eight double knockout and eight control mice were analyzed using micro-X-ray computerized tomography and a Small Animal Physiological Monitoring system. Tissues and samples from these mice were analyzed using standard histological and Molecular Biology methods and the significance of the results analyzed using a Student´s T-test.

**Results:**

The very few double knockout mice surviving up to adulthood display clear craniofacial abnormalities reminiscent of those seen in RASopathy mouse models, as well as thrombocytopenia, bleeding anomalies, and reduced platelet activation induced by thrombin. These surviving mice also present heart and spleen hyperplasia, and elevated numbers of myeloid-derived suppressor cells in the spleen. Mechanistically, we observed that these phenotypic alterations are accompanied by increased KRAS-GTP levels in heart, platelets and primary mouse embryonic fibroblasts from these animals.

**Conclusions:**

Our data uncovers a new, previously unidentified mechanism capable of triggering a RASopathy phenotype in mice as a result of the combined removal of HRAS and NRAS.

**Supplementary Information:**

The online version contains supplementary material available at 10.1186/s12964-024-01717-4.

## Introduction

Germline mutations affecting different *RAS* genes or other components of RAS-dependent signaling pathways are linked to a broad group of familial developmental syndromes [1, 2], including, among others, Noonan syndrome, Costello Syndrome, Cardio-Facio-Cutaneous, Neurofibromatosis, Noonan syndrome with Multiple Lentigines, Legius Syndrome, Capillary Malformation-ArterioVenous Malformation and Autosomal Dominant Intellectual Disability type-V [1, 2] that are collectively named RASopathies. It is generally accepted that the *gain-of-function* mutations found in RASopathies result in milder Ras signaling hyperactivation than the somatic mutations observed in sporadic cancers [2–6]. Many of these syndromes share a variety of external symptoms including craniofacial dysmorphism, neurocutaneous abnormalities [2], cardiovascular alterations, thrombocytopenia and coagulation defects [7, 8] as well as increased risk of tumorigenesis [3, 9] and autoimmune disease [8], developmental delay, learning disabilities, and short stature [2, 4].

Although they may share common phenotypic features, different mutations in the same gene do not necessarily lead to the manifestation of similar resulting symptoms. Furthermore, the intensity of these phenotypic symptoms may rank from mild, with almost normal life conditions, to severe, with life-threatening complications [10]. Such variabilities can also be observed and recreated in mouse models of human RASopathies. For instance, a mouse strain harboring the *KRAS*^P34R^ substitution, found in Cardio-Facio-Cutaneous patients [11], shows lung maturation delay and perinatal lethality but milder heart and craniofacial defects, whereas the *KRAS*^T58I^ mutation causes profound craniofacial abnormalities, heart and spleen hyperplasia and increased levels of myeloid cells [12]. Likewise, *PTPN11*^D61G^ mouse models of Noonan Syndrome develop cardiac defects in a gene-dosage-dependent manner [13], with more severe phenotypes in mice homozygous for the D61G substitution and milder defects in heterozygous mice [13]. It has also been shown that some RASopathy patients do not harbor mutations in genes coding components of RAS/MAPK pathway but instead show haploinsufficiency of the RAS-responsive element binding protein 1 (*RREB1*) [14], a zinc finger responsible of controlling H3K4 methylation of various MAPK pathway genes [14].

Of note, besides the mentioned cardio-facio-cutaneous phenotypes shared by many Rasopathy patients, the presence of perinatal complications not attributable to cardiovascular defects, but involving respiratory distress, retardation of normal alveolar development, bronchopulmonary dysplasia, etc., has also been documented in about 53% of reported cases [11, 15–17]*.*

We showed previously that HRAS^KO^/NRAS^KO^ double knockout (DKO) mice are viable [18] but exhibit very elevated rates of perinatal lethality due to respiratory failure [19]. In this report, we show that simultaneous removal of both HRAS and NRAS in mice leads to the generation of a RASopathy-like phenotype in the very small percentage of adult animals that are able to survive the perinatal period and reach adulthood in the absence of both HRAS and NRAS. Interestingly, the phenotype of these adult, surviving HRAS/NRAS^DKO^ mice shares common features with previously reported mouse RASopathy models, including (i) cranial, lung and heart abnormalities, (ii) hematologic disorders, thrombocytopenia and increased bleeding times, and (iii) increased intracellular levels of KRAS-GTP and activated pERK. Our data identifies the concomitant ablation of HRAS and NRAS as a new molecular alteration capable of triggering the development of RASopathy phenotypes in mice through a mechanism involving the hyperactivation of KRAS-dependent signaling*.*

## Materials and methods

### Animal care, genotyping, monitorization and statistical analysis

Laboratory mice were managed and handled according to European Communities Council Directive of 20 March 2015 (ECC/566/2015), and Spanish guidelines (RD53/2015) for the use and care of animals in research. All NRAS and HRAS knockout strains were maintained on pure C57Bl/6 background and kept on a 12 h light/dark cycle. Mice were genotyped by PCR analysis of genomic DNA isolated from mouse tails using specific primers for the control (CT) or targeted alleles of HRAS or NRAS as described in [19]. To reduce genetic heterogenicity, double heterozygous littermates were used as controls to the DKO mice; as already described, they are undistinguishable from WT mice [19]. Mice were weighted before the experiments and monitored with a Small Animal Physiological Monitoring system (Harvard Apparatus) following manufacturer’s indications. All the results from the two groups compared in this study were analyzed using a Student´s T-Test.

### Micro-CT scans

8-months-old male mice were anesthetized and imaged using the SuperArgus Micro-CT (SEDECAL, Madrid (Spain)). Images were taken with 720 plane projections, with 100 ms exposure time per projection, X-ray energies of 45 kVp, and 400 µA. Images were then reconstructed and converted to 3D images (Micro-CT SEDECAL ACQ). Cranial measurements were obtained as shown in Additional file 1 and as previously described [12].

### Peripheral blood collection

To monitor hematological parameters, blood samples were taken from the submandibular sinus at 8-months of age into microvette EDTA-coated tubes (Sarstedt Inc., Nümbrecht, Germany) and analyzed using a HEMAVET 950 (Drew Scientific Inc., Miami Lakes, FL, USA) monitoring the following parameters: platelet number, medium platelet volume, platelecrit, mean platelet component, total white blood cells, red blood cells, neutrophils, lymphocytes, monocytes, eosinophils, and basophils.

For platelet experiments, blood samples from the submandibular sinus were collected into tubes containing sodium citrate as anticoagulant (Sarstedt Inc., Nümbrecht, Germany).

### Platelet counting by flow cytometry

Platelet numbers were counted as previously described [20]. Briefly, blood (15 µl) was washed with 1 ml Tyrode’s Buffer (130 mM NaCl, 10 mM Trisodium citrate, 9 mM NaHCO_3_, 6 mM dextrose, 0.9 mM MgCl_2_, 0.81 mM KH_2_PO_4_, 10 mM Tris pH 7.4) and cells were resuspended in 800 µl Tyrode’s Buffer supplemented with 3% Bovine Serum Albumin (BSA). 30 µl of the previous cell solution were collected and incubated 15 min at RT with 1 µl anti-mouse CD41-FITC antibody (eBiosciences 11–0411-82) specific for platelets. Samples were washed with 500 µl Tyrode’s Buffer and centrifuged at 1300 g for 5 min. Pellets were resuspended in 1 ml Tyrode’s Buffer and 50 µl of the solution were analyzed in a BD Accuri™ C6 cytometer. Platelet concentration (per ml) was calculated from the number of platelets (CD41 positive events) in the 50 µl sample.

### Platelet purification

Blood obtained as described above was processed to obtain the platelet-rich plasma (PRP) as previously described [21] by centrifuging the blood twice for 4 min at 100 g. Platelets were then isolated from PRP after a 5 min centrifugation step at 1200 g. Before proceeding, platelets were resuspended in Tyrode’s Buffer and allowed to rest at room temperature (RT) for at least 1 h.

### Platelet activation assays

Platelets were activated with different agonists: Thrombin (Thr, Thrombin from bovine plasma, SIGMA T7513-50UN) 0.5 and 1 U; Adenosine 5’-diphosphate (ADP, SIGMA A4386) 10 µM; Collagen-related peptide (CRP, Peptide Synthetics) 2 µg/ml and 5 µg/ml; and Phorbol-myristate-acetate (PMA, SIGMA P1585) 0.2 µM and 2 µM, and platelet activation was determined by measuring the amount of activated integrin αIIbβ3, as well as by the exposure of P-selectin from the α-granules on the platelet surface as previously described [21]. 50 µl of blood were washed with Tyrode’s Buffer and resuspended in 1 ml Tyrode’s Buffer, 30 µl of diluted blood was incubated in presence of the aforementioned agonists for 15 min at RT, in presence of anti-mouse CD41-APC (eBiosciences 17–0411-82), Fibrinogen-Alexa Fluor 488 (Molecular Probes, F13191), anti-mouse αIIbβ3-PE (JON/A clone) (Emfret M023-2), and anti-CD62P-PE (BD Biosciences, 550561) antibodies. The percentage of activated platelets was assessed using a BD Accuri™ C6 cytometer.

### Immunofluorescence detection in platelets

Isolated platelets were allowed to rest at RT for at least 1 h, and if necessary, treated with Thrombin 0.5 or 1 U for 15 min. Then, platelets were fixed using 4% paraformaldehyde (PFA, PanReac BioChem, 252931.1214) for 15 min at RT and centrifuged 10 min 3200 g. Platelets were resuspended in Tyrode’s buffer and plated onto poly-L-Lysine-coated coverslips for 50 min. Attached platelets were then washed 3 × 10 with PBS, and 10 in Phosphate Buffered Saline (PBS) with Triton (0.1%) and blocked during 1 h at RT in 1X PBS containing 0.1% Triton, 5% BSA and 2% goat / donkey serum. Slides were incubated with primary antibodies β-Tubulin (1:1000, SIGMA, T5293), P-Selectin (1:200, Santa Cruz Biotechnologies, sc6941) or Cleaved Caspase-3 (CC3) (1:400, Cell Signaling, 9661) and incubated in PBS containing 0.1% Triton, 2% BSA and 2% goat / donkey serum o/n at 4 ºC. After 3 × 10 min PBS washes and 10 min in PBS-Triton (0.1%), sections were incubated with the secondary antibodies (1:500), goat anti-mouse Alexa 647, goat anti-rabbit Cy3, donkey anti-goat Cy3, and counterstained with nuclear DAPI (Sigma) and Alexa 488-Phalloidin, in the aforementioned antibody solution during 1 h at RT. Finally, coverslips were washed 3 × 10 min with PBS and mounted with ProLong Diamond antifading reagent (Life Technologies, P36970) and examined using a confocal microscope (Leica TCS SP8).

### Tail-bleeding assays

Tail-bleeding assays were performed as described before [21]. Briefly, mice were anesthetized with isofluorane. A section of 5 mm of the tail tip was transected and the tail was immersed in pre-warmed PBS at 37 ºC to avoid vasoconstriction. Bleeding time was noted when the blood flow ceased. To avoid the death of the animal, the experiments were stopped after a maximum of 8 min of bleeding.

### RAS-GTP or RAP1-GTP Pull-down assays

To monitor RAS and RAP1 activation, RAS [22] and RAP1 [21] pulldowns were performed as previously described, with minor modifications for heart tissue from 8-month old mice or isolated platelet from peripheral blood.

For heart RAS-GTP pull-downs, the tissue was homogenized in 800 µl of 1X magnesium lysis buffer (MLB) (25 mM HEPES, 150 mM NaCl, 1% IGEPAL CA-360, 10 mM MgCl_2_, 1 mM EDTA, 2% glycerol, 5 mM Na_3_VO_4_, 1 mM PMSF, EDTA-free protease inhibitor cocktail Complete®, 1 tablet/50 ml), using the GentleMACS dissociator, followed by a 10 min centrifugation at 1300 rpm at 4 ºC. Supernatants were collected and the protein concentration measured. 500 µg of protein extracts were incubated with 150 µl of beads attached to GST-RAF-RBD during 30 min at 4ºC with gentle rotation. The samples were then washed twice in 1X MLB. After the last centrifugation, supernatants were discarded, and pellets were resuspended in 60 µl of 4X Loading buffer (LB) and quickly frozen at -80ºC until use. For total protein analysis, 100 µl of heart lysates were mixed with 20 µl of 6X LB and frozen at -80 ºC until use. Finally, 20 µl of each pull-down assay sample and 50 µg of total protein lysates were loaded into 12% acrylamide gels, and levels of KRAS·GTP and Total-KRAS (1:500, SIGMA, WH003845M1), panRAS-GTP and total panRAS (1:1000, Merck Millipore, 005–516), as well as pERK (1:1000, Cell Signaling, 9101) and total ERK (1:5000, Cell Signaling, 4696) were analyzed by western blot assays with the incubation of the membranes with specific primary antibodies overnight at 4 °C. Membranes were scanned on an Odyssey Imaging System and quantitation performed using Image J 1.53c software (National Institutes of Health, USA).

For platelet RAS-GTP and RAP1-GTP pull-down assays, isolated platelets were allowed to rest at RT for 1 h prior to stimulate them with 0.5 or 1 U of Thrombin for 2 min at RT. Then, platelets were lysed in 50 µl of either 2X MLB buffer (RAS-GTP pull-down) or 2X RIPA buffer (RAP1-GTP pull-down) so that the final volume was 100 µl. 85 µl of the lysates were incubated with 150 µl of beads attached to either GST-RAF-RBD (RAS-GTP pull-down) or GST-RALGDS (RAP1-GTP pull-down) during 30 min at 4ºC with gentle rotation, and the samples were then washed twice in 1X MLB. After the last centrifugation, supernatants were discarded, pellets were resuspended in 20 µl of 2X LB and quickly frozen at -80ºC until use. For total protein analysis, 15 µl of platelet lysates were mixed with 5 µl of 4X LB and frozen at -80 ºC until use. Finally, 20 µl of each pull-down assay sample and of total protein lysates were loaded into 12% acrylamide gels, and levels of KRAS·GTP and total-KRAS (1:500, SIGMA, WH003845M1), panRAS-GTP and total panRAS (1:1000, Merck Millipore, 005–516), RAP1-GTP and total-RAP1 (1:1000, Santa Cruz Biotechnologies, sc-65), pERK (1:1000, Cell Signaling, 9101) and total ERK (1:5000, Cell Signaling, 4696) were analyzed by western blot assays with the incubation of the membranes with specific primary antibodies overnight at 4 °C. Membranes were scanned on an Odyssey Imaging System and quantitation performed using Image J 1.53c software (National Institutes of Health, USA).

### Megakaryocyte population assays in bone marrow and spleen

Bone marrow (BM) from 8-months-old mice were extracted as previously described [20] using 20 G needles from femur and tibia and placed into cold PBS. Spleen from 8-months old were mechanically disrupted using a 70 µm filter in cold PBS. Erythrocytes were lysed using 2 ml Red Blood Cell lysis buffer (RBC) for 2 min (BM samples) or 12 min (Spleen samples) on ice. Samples were centrifuged 5 min at 1300 rpm, resuspended in PBS + 3% BSA and labelled with the specific antibodies for flow cytometry: PE anti-mouse CD61 (eBiosciences, 12–0611-82), FITC anti-mouse CD42b (Emfret Analytics, M041-1), and FITC anti-mouse CD41 (eBiosciences, 11–0411-82) for 20 min in ice. The percentage of total megakaryocytes (CD41/CD61) and mature megakaryocytes (CD61/CD42) were assessed using a BD Accuri™ C6 cytometer.

### Myeloid derived Suppressor Cell (MDSC) population in peripheral blood and spleen

MDSC cell population was analyzed in both peripheral blood samples and spleen homogenates by flow cytometry from 8-months old animals. Peripheral blood was collected into sodium citrate tubes (Sarstedt Inc., Nümbrecht, Germany), and spleen was mechanically disrupted using 70 µm filters. Erythrocytes were lysed using RBC buffer for 20 min (peripheral blood samples) or 12 min (Spleen lysates) in ice. Samples were centrifuged 5 min at 1300 rpm and resuspended in PBS + 3% BSA and further labelled with the specific antibodies for flow cytometry: FITC anti-mouse Ly-6G/ly-6C (Gr1) (BioLegends, 108,406) and PE anti-mouse CD11b (BioLegends, 101,207) for 20 min on ice. The percentage of MDSC cell population (GR1/CD11b) was assessed using a BD Accuri™ C6 cytometer.

### Tissue collection and preparation

Adult animals were euthanized with an isoflurane (IsoFlo®, Esteve) overdose. Heart, spleen, lungs were harvested, weighted and washed in PBS prior to either snap-freeze in dry ice for RAS-GTP pull-down assays or for quantitative real-time PCR (RT-qPCR), or fixation with 4% PFA (PanReac BioChem, 252,931.1214) at 4 ºC for three days before dehydration and paraffin embedding as previously described in [23]. 

### Histology and immunohistochemistry

Three-micrometer sections were used for tissue staining with Hematoxylin–Eosin (H&E) according to standard procedures, and to detect pERK1/2 (1:500, Cell signaling, 9101) activation in heart tissue slices, avidin–biotin-peroxidase immunohistochemistry were performed following the protocol as previously described [19]. Immunostained sections were counterstained with diluted Harris hematoxylin during 30 s, washed in distilled water and dehydrated and mounted following standard procedures. Pictures were acquired under a light microscope (Olympus BX51 with Olympus DP70 camera). Quantification was performed in a semi-automatic way using R-Statistical Software [24] (version 4.2.1, 2022–06-23 ucrt) and a R-script that discriminates between the Immunohistochemistry signal (brown pixels), correct for the background and calculates the signal by means of brown pixels / total pixels. The following R-packages were used to read and write tiff images [25], read and write jpeg images [26], and to measure the colors present in the image, and the relative proportion of each color in the image [27]. Ten 40 × pictures per animal were analyzed.

### RAS/MAPK activation assays in mouse embryonic fibroblasts (MEFs) upon EGF stimulation

Controls and HRAS/NRAS-double null MEFs were isolated from E13.5 embryos as described [22] and analyzed as primary, low passage (< 6) culture cells. Cells were serum-deprived for 12 h and stimulated with 50 ng/ml of epidermal growth factor (EGF) for 2, 5, 10, 15, 30, 60, 120 and 240 min at 37 ºC. Then, cells were lysed in 1X MLB and RAS-GTP pull-down and western blot assays were performed as described above for platelets.

## Results

### Surviving adult HRAS/NRAS^DKO^ mice are smaller and show craniofacial dysmorphia

**Fig. 1:**
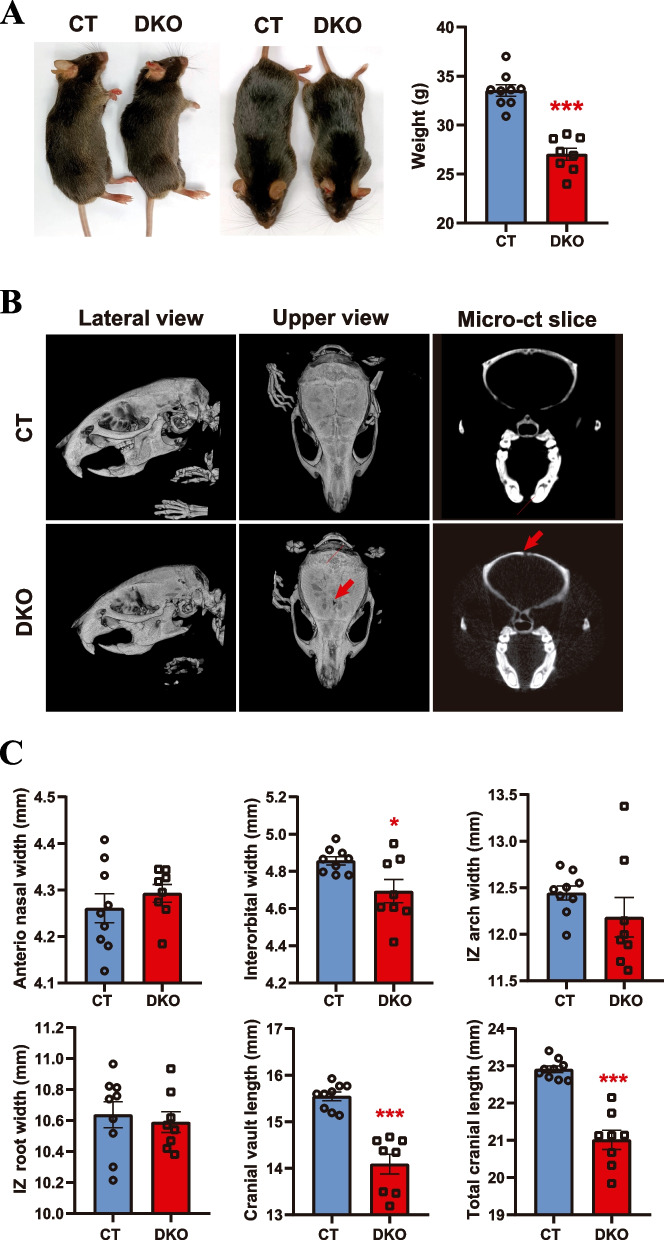
Reduced size and craniofacial dysmorphia of adult HRAS/NRAS^DKO^ mice. **A**. 8-month-old DKO animals have a lower body size and weight compared to wild-type controls (CT). Data is represented as the mean ± S.E.M. *** *p* < 0.001, *n* = 8–9. **B.** Representative 3D reconstructions of micro-CT scan images of DKO and CT 8-month-old mice. Red arrows point to a gap in the cranial sutures found in the DKO skulls. **C**. Standard skull measurements in millimeters (mm) from micro-CT images showing marked alterations in the cranial structure of DKO animals. Data represented as the mean ± S.E.M. *** *p* < 0.001, * *p* < 0.05 *n* = 8–9

We previously showed that newborn HRAS^KO^/NRAS^KO^ double-null (DKO) mice are smaller than their littermates [19]. To analyze whether this phenotype is maintained in the very scant surviving adult DKO animals, we weighted 8-month-old DKO mice, observing that they still remained smaller than the controls (CT), displaying a significantly reduced body weight (DKO 27.02 ± 0.62 g *vs* CT 33.53 ± 0.57 g) (Fig. 1A). Additionally, the external morphology of the head and snout of the DKO mice looked significantly flattened as compared to the controls. This was further confirmed by micro-X-ray computed tomography (micro-CT) of our DKO adult mice, revealing a profound cranial dysmorphia (Fig. 1B), reminiscent of that observed in mouse RASopathy models [7, 12, 28, 29]. Of note, the survivor DKO mice had also an abnormal cranial suture closure (Fig. 1B, red arrows), a feature described in a Noonan mouse model mechanistically linked to increased ERK activity [28]. Consistent with the cranial dysmorphia, the DKO animals showed also significant reduction in several cranial measurements (Additional file 1), including interorbital width, cranial vault length, and total cranial length as compared to the CT mice (Fig. 1C).

### HRAS/NRAS^DKO^ adult mice show heart enlargement and related physiological alterations

**Fig. 2:**
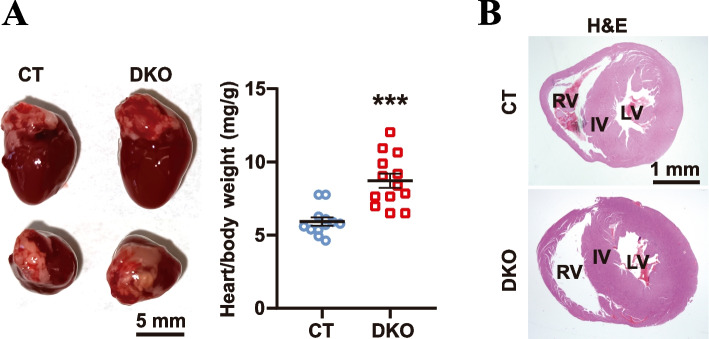
Heart enlargement in HRAS/NRAS^DKO^ adult mice. **A.** Representative lateral and top views of hearts from DKO and control animals, and measurements of the heart/body weight ratios for both genotypes. Scale bar 5 mm. Data is represented as the mean ± S.E.M. *** *p* < 0.001, *n* = 12–13. **B**. Hematoxylin–Eosin (H&E) of transversal heart sections. Interventricular wall (IV), right ventricle (RV), and left ventricle (LV) are indicated. Scale bar 1 mm

Physiological analyses of heart and lung function in our surviving, adult DKO mice showed that, consistent with our previously described observations [19], these animals exhibited significantly reduced levels of oxygen saturation in blood, although no changes in heart or breathing rates were detected (Additional file 2A). However, the respiration of the DKO animals was more altered in comparison with the controls, but no apparent alterations were observed in the electrocardiograms (Additional file 2B). Although no changes were detected in heart rate, the DKO adult mice displayed heart hyperplasia with an increased ratio of heart/body weight (Fig. 2A). Histological analyses of the heart also showed thickening of the walls of this organ (Fig. 2B), a feature resembling a common symptom found in human RASopathy patients [30, 31] as well as in some reported mouse RASopathy models [12, 32].

### Adult HRAS/NRAS^DKO^ mice show splenomegaly and increased numbers of GR1 + /CD11b + myeloid-derived suppressor cells

**Fig. 3:**
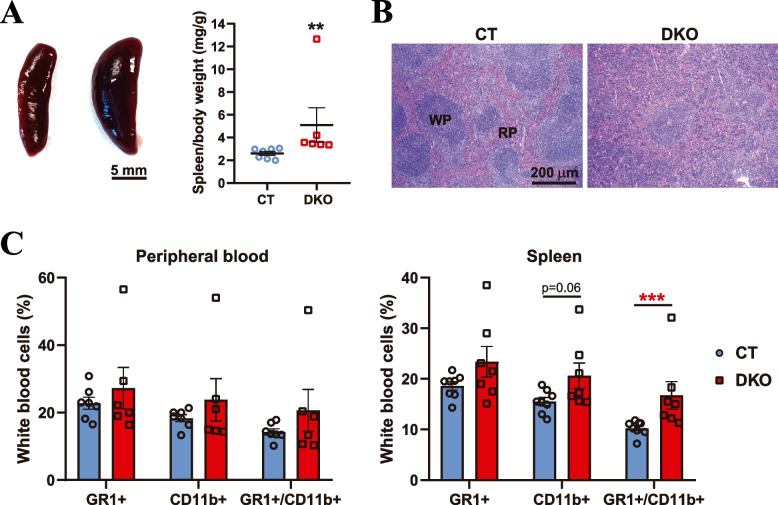
HRAS/NRAS^DKO^ mice show splenomegaly and increase of the GR1 + /CD11b + myeloid cell population. **A.** Representative spleens and analysis of spleen/body weight ratios of the indicated genotypes. Scale bar 5 mm. Data is represented as the mean ± S.E.M. ** *p* < 0.01, *n* = 6–7. **B.** H&E of spleen sections showing an aberrant tissue structure in the DKO animals. White pulp (WP) and Red pulp (RP) are indicated. Scale bar 200 µm. **C.** Flow cytometry analysis of GR1 + , CD11b + and Myeloid-Derived Suppressor Cells (MDSCs, GR1 + /CD11b +) cells in peripheral blood (left) and spleens (right). Data is represented as the mean ± S.E.M. *** *p* < 0.001, *n* = 6–8

Many RASopathy patients develop splenomegaly and have increased risk of developing myeloproliferative disorders [13, 33, 34]. Likewise, our analysis of 8-month-old DKO animals showed enlarged spleens, with some of them developing severe splenomegaly (Fig. 3A). Histopathological analyses of the spleen revealed also white pulp (WP) hyperplasia (Fig. 3B). In concordance with these observations, our analysis of peripheral blood of DKO adult mice showed (i) increased white cellularity in circulating blood but did not display visible changes in red blood cells (Additional file 3), as well as (ii) augmented content of Myeloid-Derived Suppressor Cells (MDSCs, GR1 + /CD11b + , Fig. 3C). These increases were more significant in the spleen (Fig. 3C), mainly due to an expansion of the population of GR1 + (neutrophils) and CD11b + cells (monocytes, macrophages, neutrophils, dendritic cells, natural killer cells, and particular subsets of B and CD8 + T cells).

### HRAS/NRAS^DKO^ adult mice have decreased platelet cell count along with coagulation defects

**Fig. 4:**
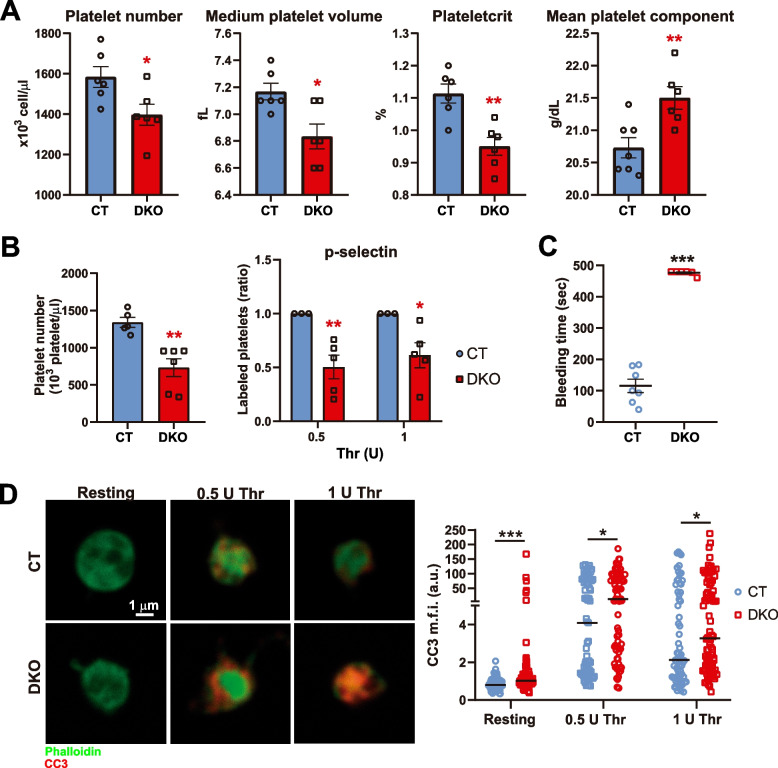
HRAS/NRAS^DKO^ mice show thrombocytopenia, lower platelet activation, increased bleeding and enhanced platelet apoptosis. **A.** Platelet number, medium platelet volume (fL, femtolitre), plateletcrit and mean platelet component (g/dL, gram/deciliter) analyses measured by means of HEMAVET 950. Data is represented as the mean ± S.E.M. * *p* < 0.05, ** *p* < 0.01, *n* = 6. **B.** CD41-labelled platelets counted by flow cytometry (left) and platelet activation upon Thrombin (Thr) stimuli (0.5/1 U) measured by flow cytometry as p-selectin translocation from intracellular granules to the external membrane (right). Data is represented as the mean ± S.E.M. For the platelet activation, each experiment (3 different experiments, 2 controls in each set of experiments) was relativized to the controls (CT) mean, * *p* < 0.05, ** *p* < 0.01, *n* = 5–6. **C.** Tail bleeding assays showed significantly increased bleeding times for DKO adult mice. Data is represented as the mean ± S.E.M. *** *p* < 0.001, *n* = 6–7. **D.** Immunofluorescence analysis of platelet apoptosis before and after stimulation with 0.5 or 1 U of Thrombin (Thr). Cleaved caspase-3 (CC3, red) and phalloidin (green). Scale bar 1 µm. Levels of CC3 mean fluorescence intensity (m.f.i., a.u., arbitrary units) in each platelet, quantified using ImageJ (NIH). Data is represented as the median of CC3 fluorescence from 85–116 platelets from 5 CT and 5 DKO animals. * *p* < 0.05, *** *p* < 0.001

Since many human RASopathies patients suffer defects in clotting and platelet physiology [35], we also analyzed blood coagulation in our DKO mice. A significant reduction in platelet number and cell volume, as well as a lower plateletcrit, were clearly detected in peripheral blood of our DKO adult animals (Fig. 4A). Additionally, DKO blood samples showed an increased mean platelet component (an indirect measurement of platelet activation), indicating decreased platelet activity (Fig. 4A). To further validate those results, we specifically labelled platelets with CD41 marker by flow cytometry and detected lower platelet counts (thrombocytopenia) in peripheral blood of DKO adult animals (Fig. 4B). Furthermore, a reduced platelet activation upon stimulation with thrombin was also observed in DKO samples (Fig. 4B). Although no statistically significant changes in platelet activation were obtained under our experimental conditions when using other activators (Additional file 4), a trend towards lower platelet activation was also observed when platelets were stimulated with collagen-related peptide (CRP) (Additional file 4). Consistent with this, when clotting was analyzed by means of a tail-bleeding assay, the DKO animals exhibited increased bleeding times (Fig. 4C). However, when we analyzed the content of secretory granules containing second-line platelet activation, we did not observe changes between CT and DKO samples regarding the number of granules per platelet area (Additional file 5).

We also investigated the levels of platelet activation in the DKO mice by measuring RAP1 activation, a critical event in platelet activation [36]. RAP1-GTP levels upon thrombin stimulation for 2 min were comparable between CT and DKO platelets (Additional file 5), suggesting that the lower platelet activation and increased bleeding may be caused by RAP1-independent mechanisms.

### Platelet alterations in HRAS/NRAS^DKO^ mice are associated with increased levels of apoptosis

To get further mechanistic insights/clues into the underlying cause of thrombocytopenia in our DKO animals, we studied the number of megakaryocytic (MK) platelet precursors in bone marrow and spleen. For this purpose, we measured the levels of total (CD61 + /CD41 +) (Additional file 6A, left) and mature MK (CD61 + /CD42 +) (Additional file 6A, right) populations in bone marrow and spleen, finding that there were no differences in the percentages of MK between CT and DKO mice, neither in bone marrow nor in spleen (Additional file 6). However, when cell death was analyzed by means of Cleaved Caspase 3 (CC3), we found increased levels of apoptosis in DKO platelets, both under basal resting conditions or after stimulation with Thrombin (Fig. 4D). These data suggest that the observed impairment of platelet count and function is probably related, at least in part, to increased apoptotic death rate of the platelets in the DKO animals.

### KRAS-GTP and pERK levels are elevated in heart tissue, MEFs and platelets from HRAS/NRAS^DKO^ mice

**Fig. 5:**
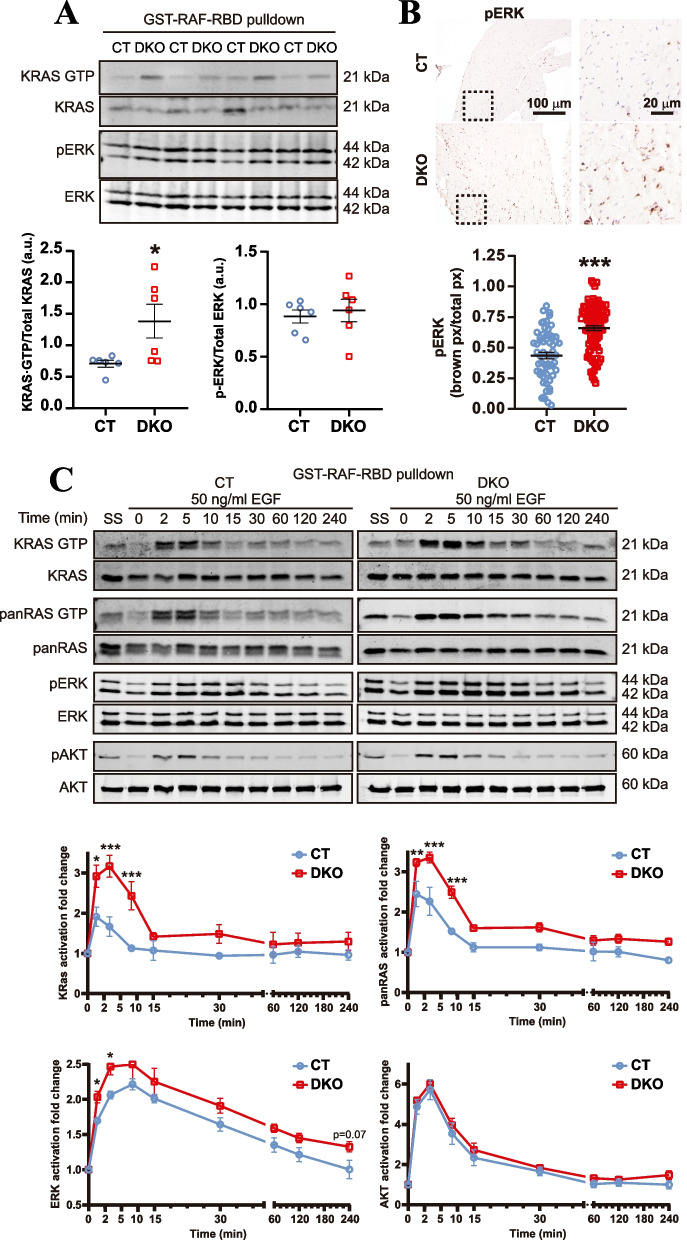
Increased KRAS and ERK activation levels in HRAS/NRAS^DKO^ heart and EGF-stimulated MEFs. **A.** Western blot of KRAS and ERK activation from pull-down heart tissue samples. Graphs show the analysis of the KRAS-GTP/total KRAS and pERK/total ERK ratios. Activation levels were quantitated as fold change relative to those measured in CT samples. Data is represented as the mean ± S.E.M. * *p* < 0.05, *n* = 6. **B.** Representative images of pERK immunohistochemistry staining of heart sections and quantification of pERK levels (ratio brown pixel relative to total pixel) showing increased ERK activation in DKO hearts. Data is represented as the mean ± S.E.M. *** *p* < 0.001. Scale bar 100 µm and 20 µm for the magnified areas. **C.** Pull-down assays and immunoblots to analyze the activation of RAS, ERK and AKT proteins in Primary mouse embryonic fibroblasts (MEFs) starved for 12 h and stimulated with 50 ng/ml of EGF for 0, 2, 5, 10, 15, 30, 60, 120 and 240 min., or steady-state samples (SS). Activation levels were calculated as KRAS.GTP/Total KRAS, panRAS.GTP/Total panRAS, pERK/ERK and pAKT/AKT ratios, and represented as the fold change relative to the values measured in starving (0) samples. Significantly increased levels of KRAS and panRAS activation were detected in DKO MEFs at 2, 5 and 10 min after EGF stimulation. Increased ERK activation was detected at 2 and 5 min. Data is represented as the mean ± S.E.M. * *p* < 0.05, ** *p* < 0.01, *** *p* < 0.001, *n* = 4

**Fig. 6:**
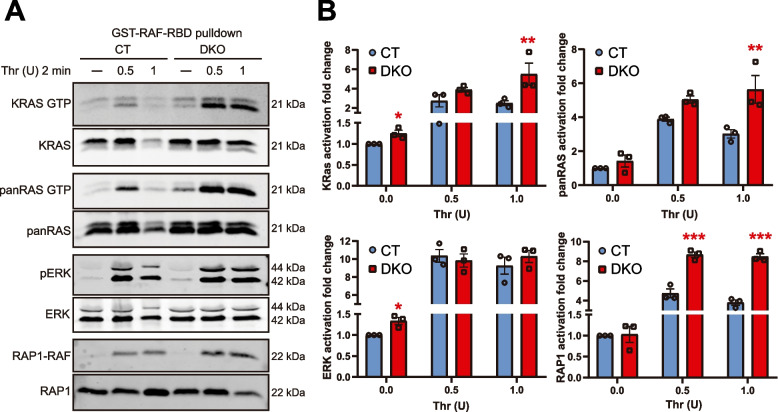
Increased KRAS·GTP and RAP1·GTP levels in HRAS/NRAS^DKO^ platelets. **A.** Representative immunoblots of protein extracts and pull-down assays using GST-RAF-RBD fusion protein from platelets stimulated with 0.5 or 1 U of Thrombin (Thr) for 2 min. Lysates were analyzed using KRAS, panRAS, pERK, ERK and RAP1 antibodies. **B.** Quantification of the activation of RAS, ERK and RAP1 proteins from Western blot assays. Activation levels were calculated as KRAS.GTP/Total KRAS, panRAS.GTP/Total panRAS, pERK/ERK and RAP1.GTP/Total RAP1 ratios, and represented as the fold change relative to those ratios measured in resting (-) CT samples. Data is represented as the mean ± S.E.M. ** *p* < 0.01, ***p < 0.001, *n* = 3

Our DKO animals show symptoms resembling those observed in human RASopathy patients, while presumably lacking the presence of the genetic, *gain of function* mutations usually associated with the development of these type of inherited syndromes. In this regard, we measured the level of activation of KRAS, the remaining canonical member of the RAS family, in our HRAS/NRAS^DKO^ animals. For this purpose, we analyzed samples from heart tissue, MEFs and platelets by means of pull-down assays using a GST-RAF-RBD (RAS Binding Domain) fusion protein and Western blot assays (Fig. 5 and 6).

Interestingly, our analysis of the DKO heart tissues uncovered a statistically significant increase of KRAS-GTP levels and a non-statistical increase in pERK levels when compared with control heart tissue extracts (Fig. 5A). ERK activation was confirmed by a significant increase in pERK levels immunologically detected in histological heart sections of the corresponding DKO and CT genotypes (Fig. 5B).

To corroborate whether the increase in RAS/MAPK activation is also observed in DKO MEFs, we stimulated CT and DKO primary MEFs (previously starved for 12 h), with EGF for different time periods up to a maximum 240 min, and performed Pull-down and Western blot assays using KRAS, PanRAS, ERK/pERK and AKT/pAKT antibodies. (Fig. 5C). Interestingly, and in concordance with our previous observations in heart tissue, DKO MEFs showed a significant increment in the levels of KRAS and ERK activation, albeit no changes in AKT activation were detected between CT and DKO samples (Fig. 5C).

Furthermore, the comparative analysis of untreated and thrombin-stimulated platelets to measure KRAS, panRAS, ERK1/2 and RAP1 activation levels (Fig. 6) revealed higher KRAS activation levels in resting and thrombin-stimulated DKO platelets, but only higher pERK levels in resting DKO platelets (Fig. 6). Interestingly, we also detected higher levels of RAP1 bound to RAF-RBD in platelets from DKO animals (Fig. 6).

Taken together, these observations suggest that the increased KRAS/MAPK signaling observed in different cells and tissues from our HRAS/NRAS^DKO^ mice provides, at least in part, a mechanistic explanation accounting for the acquisition of the RASopathy-like phenotypes that are clearly visible in the reduced percentage of HRAS/NRAS^DKO^ mice that manage to reach adulthood.

## Discussion

Germline mutations in RAS or other components of the Receptor Tyrosine Kinase-RAS-ERK signaling pathways are responsible for a group of developmental disorders, globally designated as RASopathies [1–3]. The *gain of function* mutations observed in RASopathies are thought to produce milder levels of overall signaling hyperactivation than the somatic activating mutations that are driving development of different sporadic cancers [2, 37].

Besides other phenotypic features frequently shared by different RASopathies (such as cranial, heart, and spleen abnormalities, blood coagulation defects, and thrombocytopenia, as well as increased risk of developing myeloproliferative disorders), there are various reports describing respiratory failure in newborn infants carrying different RASopathy mutations [11, 15–17, 38]. Interestingly, these RASopathy-associated respiratory failures are clearly reminiscent of our previously published observations in newborn HRAS/NRAS^DKO^ mice showing exceedingly high rates of neonatal death due to respiratory failure [19]. In this regard, here we wished to test in detail whether the very few adult HRAS/NRAS^DKO^ mice (< 7% of all born HRAS/NRAS^DKO^ animals) [19] that are able to survive and eventually reach adulthood might also display some kind of RASopathy-like phenotypic defect or clinical feature. Consistent with this hypothesis, the immature lungs of our newborn HRAS/NRAS^DKO^ animals [19] share similarities, including in particular a significantly increased rate of perinatal lethality, with a murine model of Noonan syndrome harboring a KRAS^P34R^ mutation [12], an amino acid which is known to modulate GTP hydrolysis in KRAS proteins (Bera et al. 2020). Interestingly, in this regard, it has also been recently shown that downstream MEK inhibition may ameliorate, but is insufficient, to revert pulmonary vascular disease in Noonan patients [39, 40]. Moreover, the lung tissues of our surviving, adult HRAS/NRAS^DKO^ mice present a patched phenotype, with areas of atelectasis within emphysema lesions and normal lung tissue [19] which could account for the reduced levels of oxygen saturation in blood, and the distorted respiration patterns reported in this study.

In this report, we observed that, in addition to a sizeable reduction of body weight, the HRAS/NRAS^DKO^ adult mice displayed also various craniofacial defects, involving a reduced cranial vault length and the presence of cranial suture closure abnormalities, which could probably be mechanistically linked to increased pERK signaling as previously described in a mouse model of Noonan syndrome [28]. Together with these findings, our HRAS/NRAS^DKO^ mice also displayed increased heart/body weight ratios and thickening of the cardiac walls, although their cardiac function appeared to remain normal. It is worth mentioning that, in addition to the phenotypes described in this work, our DKO mice, especially males, are infertile (unpublished data), which is a common symptom found in RASopathy patients [41].

As observed with mouse models and in our DKO mice, cardiac defects in RASopathies vary among different congenital or de novo pathogenic mutations (reviewed in [42, 43]). For example, KRAS^V14I^ animals have heart hyperplasia and aortic valve thickening with normal function [29], while KRAS^P34R^ mice exhibit a slight increase in valve areas [12], KRAS^T58I^ mice develop hypertrophic cardiomyopathy [12], and PTPN11^D61G^ animals display gene-dosage-dependent defects, with severe manifestations in homozygotes (heart failure by E13.5, pan-valvular stenosis, large septal defects) and milder defects in heterozygotes [13].

Splenomegaly and hematological defects, such as those observed in our DKO mice, are also frequent major features of RASopathies [35], and are also present in patients carrying somatic mutations responsible for RAS-associated autoimmune leukoproliferative disorder [2, 9, 44]. In both types of illnesses, the patients show increased white pulp cellularity, accompanied in some cases by an elevated number of myeloid cells in circulating blood and/or spleen [33, 34, 45–47]. This condition can eventually give rise to a benign form of myeloproliferative disorder that regresses spontaneously [13, 33, 34] or progresses toward juvenile myelomonocytic leukemia [2, 13, 34]. As with previously described RASopathies, the degree of splenomegaly and related histological alterations in our HRAS/NRAS^DKO^ animals varies from mouse to mouse. Thus, about 16% of them developed a significant increment in MDSCs and about 33% have a milder increase. On the other hand, about half of the DKO animals exhibited a similar percentage of MDSCs than Control mice in circulating blood. However, the changes in the number of MDSCs in the spleen were more evident, with all the DKO mice showing enrichment in the population of this cell type.

We also uncovered defective clotting and thrombocytopenia in our HRAS/NRAS^DKO^ mice, two features recently linked to RASopathy mutations in patients [35], as well as to a SHP2-deficient mouse model [7]. Our HRAS/NRAS^DKO^ double null animals exhibited significantly low platelet count as well as reduced platelet activation, and increased bleeding. However, neither the numbers of α-granules nor the levels of RAP1 activation were affected, and a normal number of megakaryocytes (MK) was also detected in the bone marrow and spleen of the DKO mice. Interestingly, we observed increased levels of apoptosis in DKO platelets which might be related to the reduced number and activation found in this cell type, as well as the defects in coagulation. Preliminary work from our laboratory also suggests that the increased CC3 levels are likely caused by exacerbated Ca^2+^-dependent signaling occurring in the DKO samples, both under basal conditions and upon thrombin stimulation.

Mechanistically, the increased levels of activated KRAS-GTP and pERK detected in various biological samples including MEFs, heart tissue, or isolated blood platelets, appear to be the main driving force behind the various RASopathy-like phenotypes displayed by our HRAS/NRAS^DKO^ mice. The increased level of KRAS downstream signaling is not due to augmented expression of KRAS in the DKO mice, as total KRAS levels were similar between genotypes in our immunoblots from heart, platelets or MEFs and, in addition, our prior transcriptional analyses of single HRAS^KO^ or NRAS^KO^ MEFs, as well as double HRAS/NRAS^DKO^ or RAS-less MEFs, did not show compensatory transcriptional alterations of any of the three individual canonical RAS isoforms in the absence of any of the others [48–50]*.* Thus, the observed alterations must be due to dysregulation/hyperactivation of KRAS and downstream signaling occurring in the absence of HRAS and NRAS in the cells and tissues of our DKO mice. It is worth mentioning that, opposed to ICH analysis, pERK activation in heart samples is not significantly increased. This could be due to a higher activation of pERK in a specific cell type in the heart, having normal levels in the rest, which would conceal the activation in that cell type when analyzing the organ extracts. In addition, in data not included in this work we have observed that cell size is normal in our 8 months old mice and proliferation measured with Ki67 is scarce at this stage, suggesting that hyperactivation of RAS signaling at earlier times should be responsible of the observed phenotype. This mechanistic notion about the origin of the in vivo phenotypes of the HRAS/NRAS^DKO^ mice, is indeed consistent with our observations in DKO MEFs, where we demonstrated here that EGF-stimulation resulted in significantly increased levels of activation of KRAS and ERK but showed no changes of AKT activation in comparison to CT MEFs. Likewise, the various RASopathy-like phenotypic defects related to blood clotting and hemostasis appear also to be mechanistically linked, at least in part, to the hyperactivation of KRAS signaling observed in platelets, a cellular type where KRAS regulation by microRNAs has been reported to play a relevant functional role [51]. In this regard, although ERK1/2 activation in platelets may not be regulated by RAS/RAF interaction [52], KRAS hyperactivation might be promoting apoptosis in DKO platelets by triggering excessive Ca^2+^ release from the endoplasmic reticulum [53] or through CC3 activation driven by the mitochondria [54–56].

Regarding the currently described, specific ability of KRAS to drive RASopathy phenotypes in the absence of the other two (HRAS and NRAS) canonical members of the RAS family, it is pertinent to say that although only KRAS is necessary and sufficient for development to the adult stage [18], our previous transcriptional studies have also documented the critical, specific involvement of HRAS in proliferation, NRAS in immune modulation/host defence and apoptotic responses [48, 49]; and KRAS in cell cycle progression [50, 57]. It is therefore reasonable that, although HRAS and NRAS are not critically needed for survival, their absence in the DKO is an essential mechanistic driver of the appearance of the defective, non-lethal RASopathy phenotypes described here. In this regard, our initial description of the HRAS/NRAS^DKO^ mice already noted that the number of double knockout animals resulting from crosses between heterozygous NRAS/HRAS animals was lower than expected according to Mendelian ratios [18] and our later studies have identified the defective lung development and respiratory failure of the DKO [19], indicating that the impossibility of physiological, functional interplay among the three canonical RAS members (H,N and K) in organs and tissues of the DKO mice is the ultimate origin of the defective RASopathy phenotypes observed in our HRAS/NRAS^DKO^ mice.

Whether, and how, other RAS family members may be also contributing to the phenotypic traits of adult DKO mice remains to be clarified in the future. For instance, although we have detected elevated levels of RAP1-RAF binding in DKO platelets, it has also been reported that, in some contexts of RAS/MAPK hyperactivation, RAP1 competes with RAS for RAF binding, thus lowering the overall activation of this signaling pathway [58–60]. In this regard, the increased interaction of RAP1 with RAF could also be interpreted as an unsuccessful mechanism to try and compensate for the excessive KRAS activation occurring in the DKO platelets.

## Conclusions

Altogether, the data in this report strongly support the notion that the combined ablation of HRAS and NRAS leads to increased levels of activated, KRAS-dependent signals that are then responsible for the development of the RASopathy-like phenotypes of DKO mice without the need to acquire any additional, genetic hyperactivating mutations in the signaling pathway. Future studies using some of the specific inhibitors of KRAS isoforms recently developed [61, 62], as well as additional experimental assays and sequencing analyses of DKO biological samples at the level of individual tissues or cell types may be instrumental to confirm this hypothesis.

### Supplementary Information

Additional file 1. Cranial measurements performed in our adult mice. Micro-CT scans and 3D reconstruction images indicating the different measurements described in the text.

Additional file 2. Respiratory and cardiac parameters in adult CT and HRAS/NRAS^DKO^ mice. A. Blood oxygen (O_2_) saturation, heart rate (bpm, beats per minute) and respiration rate (rpm, respirations per minute) performed on 8-month-old mice. Significantly lower levels of O_2_ were observed in DKO animals. Data is represented as the mean ± S.E.M. *** *p*<0.001, *n*=9. B. Respiration rate and amplitude (a.u., arbitrary units) measurements from anesthetized CT and HRAS/NRAS^DKO^ 8-months-old mice show an increase in both parameters in the DKO animals. Electrocardiograms (ECG, mV, millivolts) performed on CT and HRAS/NRAS^DKO^ mice of the same age under anesthesia as described in Materials and Methods.

Additional file 3. Adult HRAS/NRAS^DKO^ mice exhibit increased white blood cell cellularity. Red blood cells, white blood cells, neutrophils, lymphocytes, monocytes, eosinophils and basophils levels in circulating blood measured by means of HEMAVET 950. Data represented as the mean ± S.E.M. * *p*<0.05, *n*=6-7.

Additional file 4. Platelet activation assays using flow cytometry. Platelet activation was measured upon treatment with Collagen-Related Peptide (CRP, 2/5 µg/ml), Phorbol-myristate-acetate (PMA, 0.2/2 µM) or with Adenosine 5’-diphosphate (ADP, 10 µM) by flow cytometry methods. Data are represented as the mean ± S.E.M. For the platelet activation assays, each experiment was normalized to the mean value of the controls. CT *n*=3, DKO n = 4-6.

Additional file 5. Platelet activation is unaffected in DKO mice. Measurements of granule secretion and RAP1 activation in CT and DKO animals. A. Immunofluorescence images of platelets stained with phalloidin (green) and p-selectin (α-granules, red). Scale bar 1 µm. Graphs quantitate the median fluorescence intensity (m.f.i., a.u., arbitrary units) quantified using ImageJ (NIH) of α-granules, and platelet area. Data is represented as the median, *n*=4-6. B. GST-RALGDS RAP1 pull-down assays of platelets in resting conditions or stimulated with 0.5/1 U Thrombin (Thr) for 2 min. RAP1 activation levels were quantitated as fold change relative to those measured in resting CT samples. Data is represented as the mean ± S.E.M, *n*=5.

Additional file 6. HRAS/NRAS^DKO^ mice have normal number of bone marrow megakaryocytes. Percentages of total megakaryocytes (MK) CD41+/CD61+ (left) and mature MK CD61+/CD42+ (right) in bone marrow and spleen from adult CT and DKO animals. Data is represented as the mean ± S.E.M., *n*=6-7.

## Data Availability

Not applicable.

## Abbreviations

BM: Bone marrow
BSA: Bovine Serum Albumin
CC3: Cleaved Caspase 3
CRP: Collagen-related peptide
CT: Control
DKO: Double Knockout
EGF: Epidermal growth factor
LB: Loading buffer
MDSCs: Myeloid-derived suppressor cells
MEFs: Mouse Embryonic Fibroblasts
micro-CT: Micro-X-ray computed tomography
MK: Megakaryocyte
MLB: Magnesium lysis buffer
PBS: Phosphate Buffered Saline
PFA: Paraformaldehyde
PMA: Phorbol-myristate-acetate
PRP: Platelet Rich Plasma
RBC: Red Blood Cell lysis buffer
RBD: Ras Binding Domain
RP: Red pulp
Thr: Thrombin
WP: White pulp
WT: Wild Type
