# Supplementary material for: Combined HRAS and NRAS ablation induces a RASopathy phenotype in mice
Source: Cell Commun Signal. 2024 Jun 17;22:332. doi: 10.1186/s12964-024-01717-4 (PMC11184836; doi:10.1186/s12964-024-01717-4)
Supplement: Supplementary file 1 — Additional file 1. Cranial measurements performed in our adult mice. Micro-CT scans and 3D reconstruction images indicating the different measurements described in the text. [file 12964_2024_1717_MOESM1_ESM.pdf]

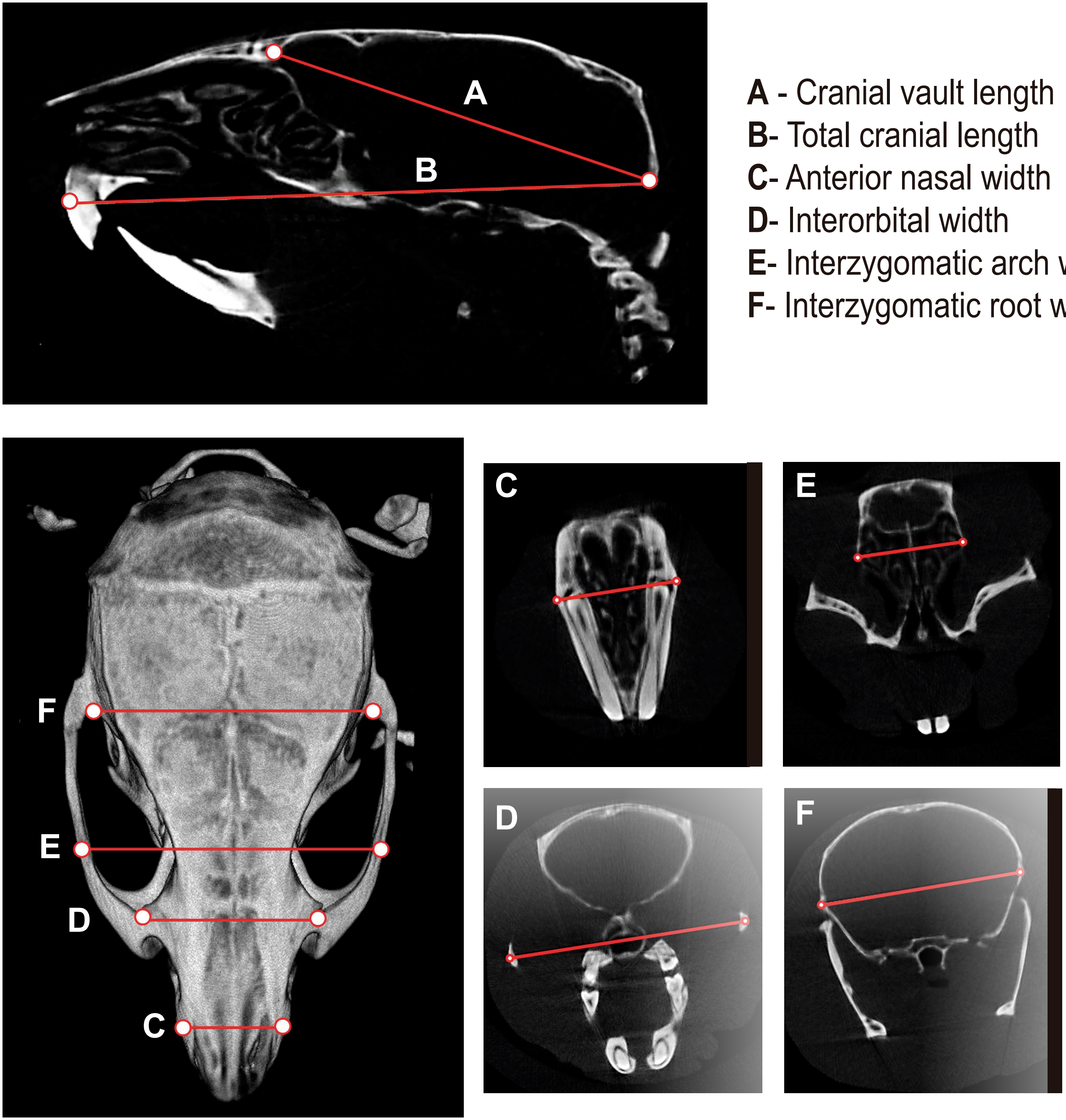

A - Cranial vault length
B- Total cranial length
C- Anterior nasal width
D- Interorbital width
E- Interzygomatic arch width
F- Interzygomatic root width
