# Supplementary figures and images for: Combined HRAS and NRAS ablation induces a RASopathy phenotype in mice
Source: Cell Commun Signal. 2024 Jun 17;22:332. doi: 10.1186/s12964-024-01717-4 (PMC11184836; doi:10.1186/s12964-024-01717-4)

A

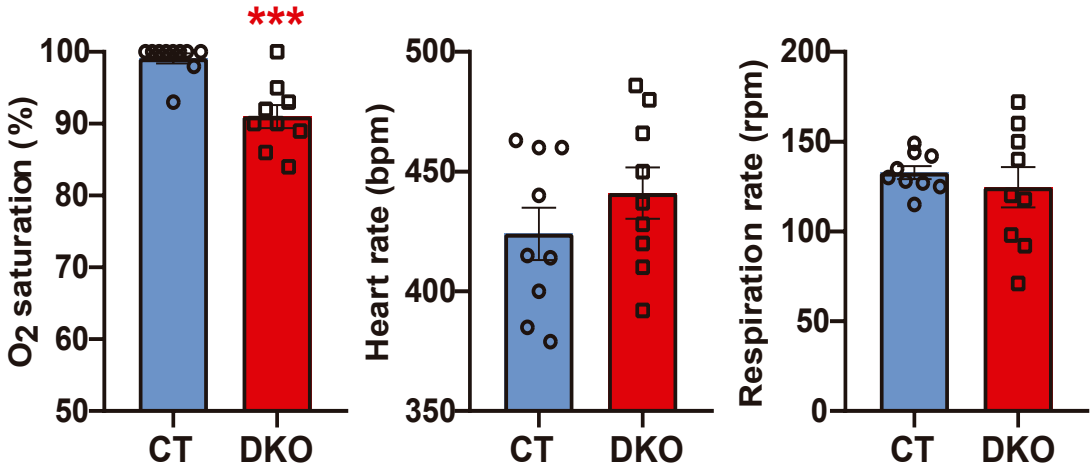

B

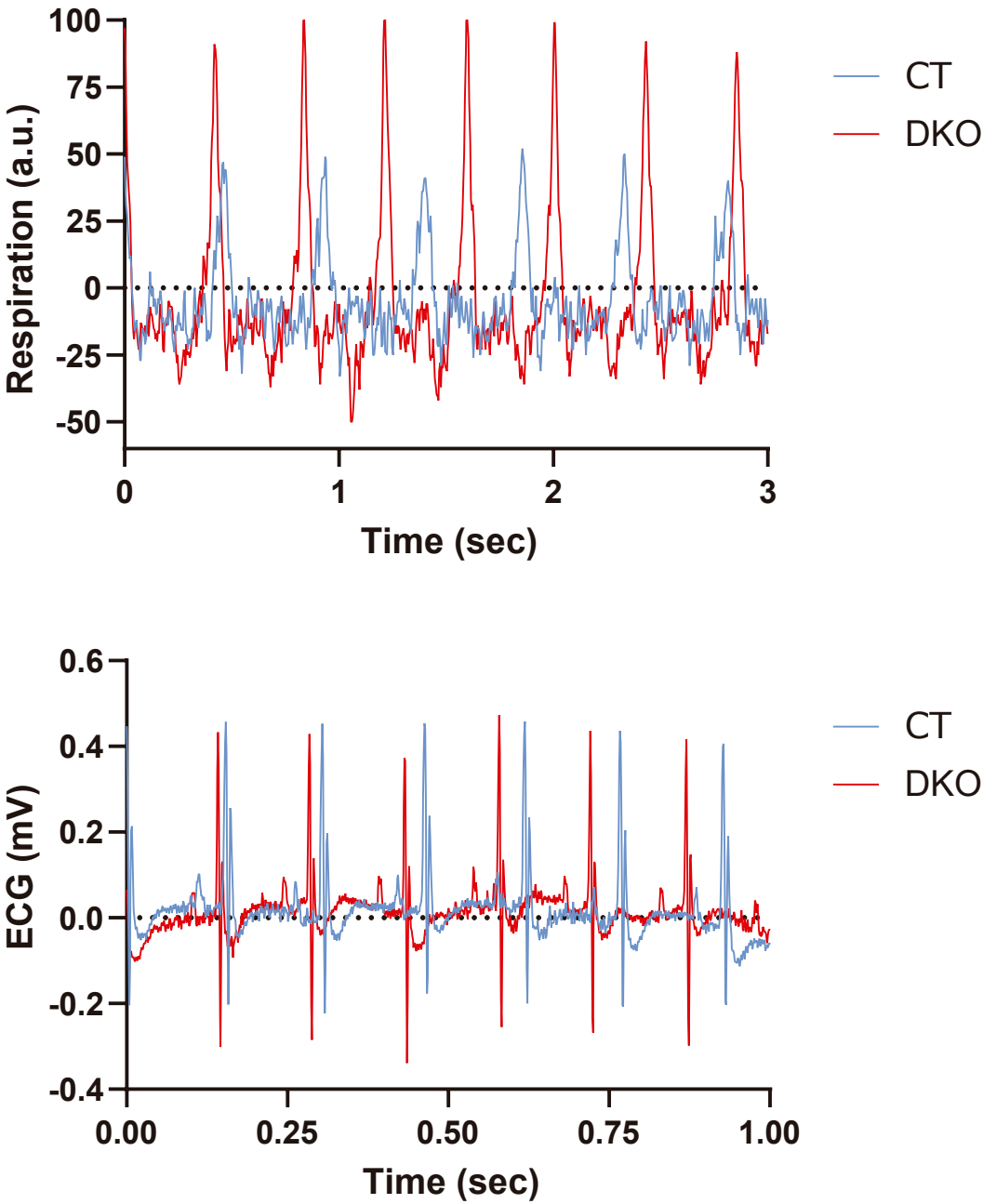

Supplement: Supplementary file 2 — Additional file 2. Respiratory and cardiac parameters in adult CT and HRAS/NRASDKO mice. A. Blood oxygen (O2) saturation, heart rate (bpm, beats per minute) and respiration rate (rpm, respirations per minute) performed on 8-month-old mice. Significantly lower levels of O2 were observed in DKO animals. Data is represented as the mean ± S.E.M. *** p<0.001, n=9. B. Respiration rate and amplitude (a.u., arbitrary units) measurements from anesthetized CT and HRAS/NRASDKO 8-months-old mice show an increase in both parameters in the DKO animals. Electrocardiograms (ECG, mV, millivolts) performed on CT and HRAS/NRASDKO mice of the same age under anesthesia as described in Materials and Methods. [file 12964_2024_1717_MOESM2_ESM.pdf]

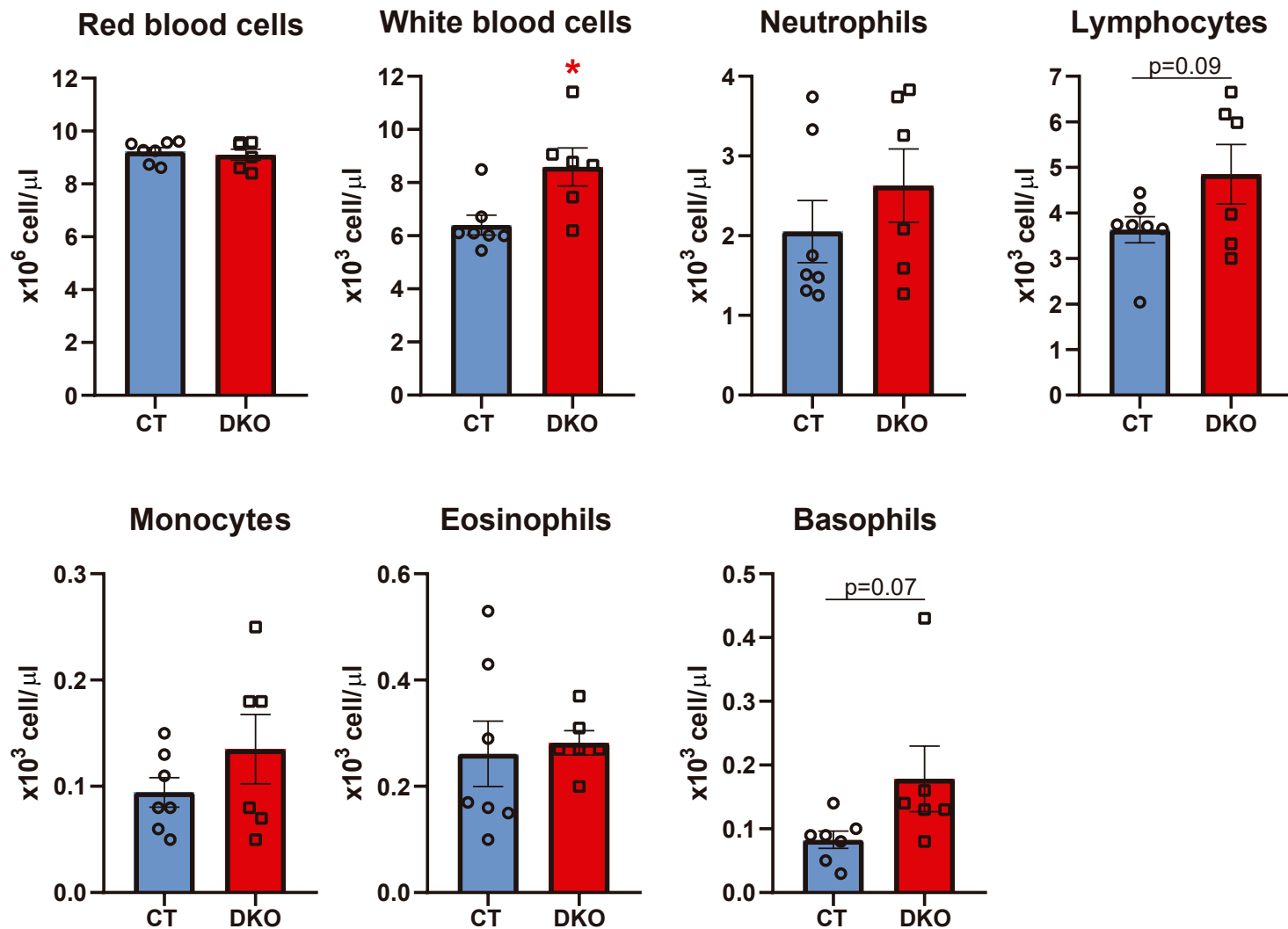

Supplement: Supplementary file 3 — Additional file 3. Adult HRAS/NRASDKO mice exhibit increased white blood cell cellularity. Red blood cells, white blood cells, neutrophils, lymphocytes, monocytes, eosinophils and basophils levels in circulating blood measured by means of HEMAVET 950. Data represented as the mean ± S.E.M. * p<0.05, n=6-7. [file 12964_2024_1717_MOESM3_ESM.pdf]

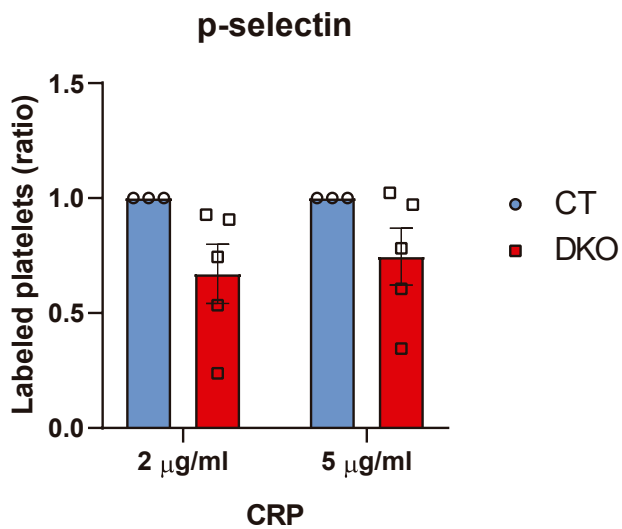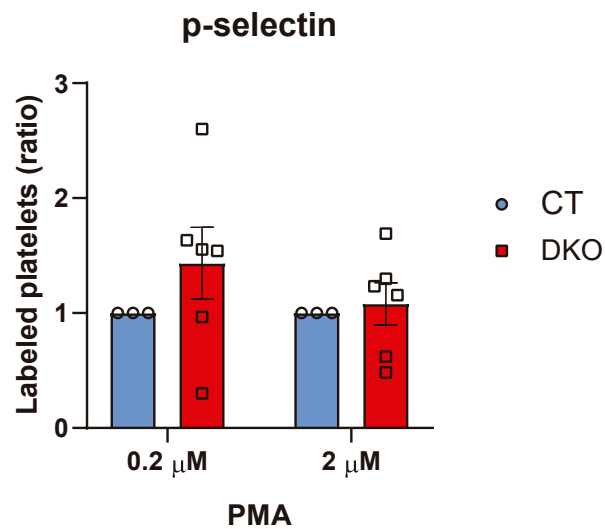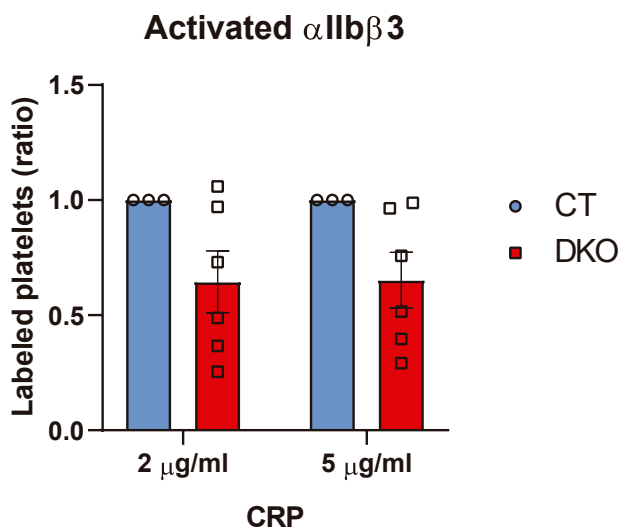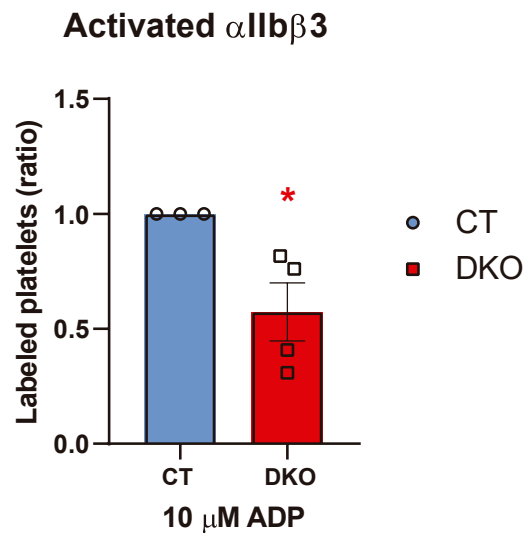

Supplement: Supplementary file 4 — Additional file 4. Platelet activation assays using flow cytometry. Platelet activation was measured upon treatment with Collagen-Related Peptide (CRP, 2/5 µg/ml), Phorbol-myristate-acetate (PMA, 0.2/2 µM) or with Adenosine 5’-diphosphate (ADP, 10 µM) by flow cytometry methods. Data are represented as the mean ± S.E.M. For the platelet activation assays, each experiment was normalized to the mean value of the controls. CT n=3, DKO n = 4-6. [file 12964_2024_1717_MOESM4_ESM.pdf]

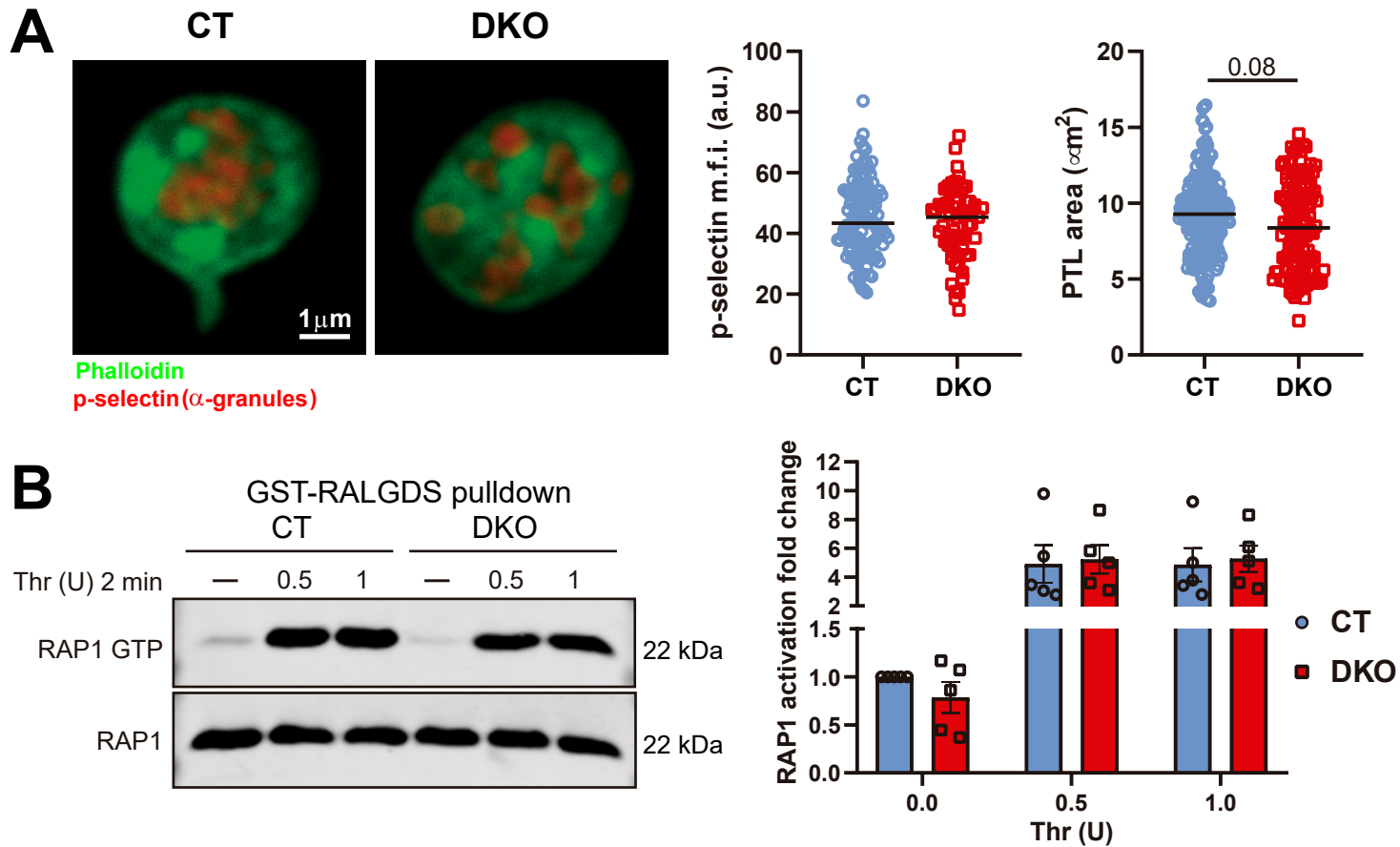

Supplement: Supplementary file 5 — Additional file 5. Platelet activation is unaffected in DKO mice. Measurements of granule secretion and RAP1 activation in CT and DKO animals. A. Immunofluorescence images of platelets stained with phalloidin (green) and p-selectin (α-granules, red). Scale bar 1 µm. Graphs quantitate the median fluorescence intensity (m.f.i., a.u., arbitrary units) quantified using ImageJ (NIH) of α-granules, and platelet area. Data is represented as the median, n=4-6. B. GST-RALGDS RAP1 pull-down assays of platelets in resting conditions or stimulated with 0.5/1 U Thrombin (Thr) for 2 min. RAP1 activation levels were quantitated as fold change relative to those measured in resting CT samples. Data is represented as the mean ± S.E.M, n=5. [file 12964_2024_1717_MOESM5_ESM.pdf]

### Bone Marrow

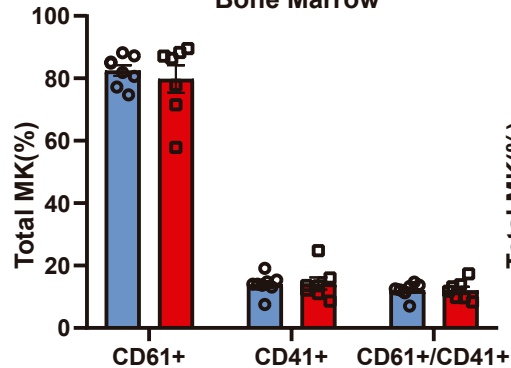

### Spleen

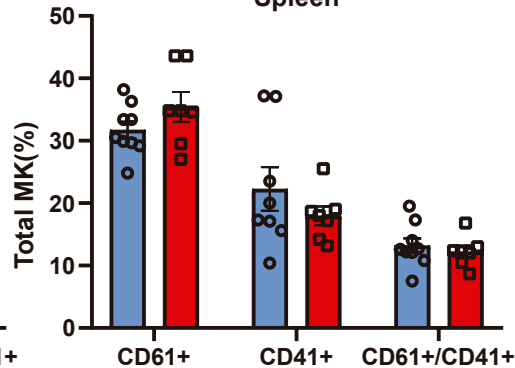

### Bone marrow

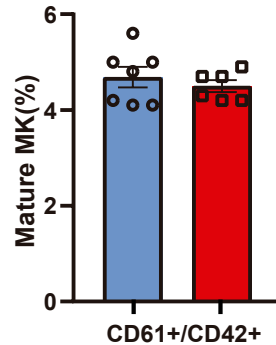

### Spleen

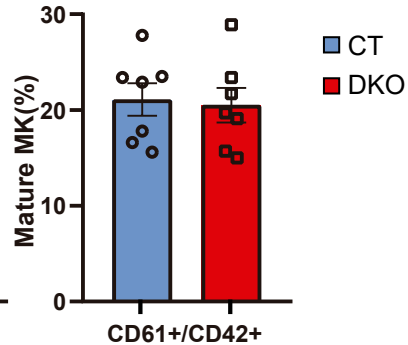

CT  
DKO

Supplement: Supplementary file 6 — Additional file 6. HRAS/NRASDKO mice have normal number of bone marrow megakaryocytes. Percentages of total megakaryocytes (MK) CD41+/CD61+ (left) and mature MK CD61+/CD42+ (right) in bone marrow and spleen from adult CT and DKO animals. Data is represented as the mean ± S.E.M., n=6-7. [file 12964_2024_1717_MOESM6_ESM.pdf]
